# Harnessing cytokinins with chemistry: new frontiers and challenges for precision plant growth control

**DOI:** 10.3389/fpls.2025.1602749

**Published:** 2025-05-29

**Authors:** Liang Cheng

**Affiliations:** ^1^ Beijing National Laboratory for Molecular Sciences (BNLMS), CAS Key Laboratory of Molecular Recognition and Function, CAS Research/Education Center for Excellence in Molecular Sciences, Institute of Chemistry, Chinese Academy of Sciences, Beijing, China; ^2^ University of Chinese Academy of Sciences, Beijing, China

**Keywords:** cytokinins, chemical modulation, plant growth regulation, bioorthogonal chemistry, crop improvement

## 1 Introduction

**Figure 1:**
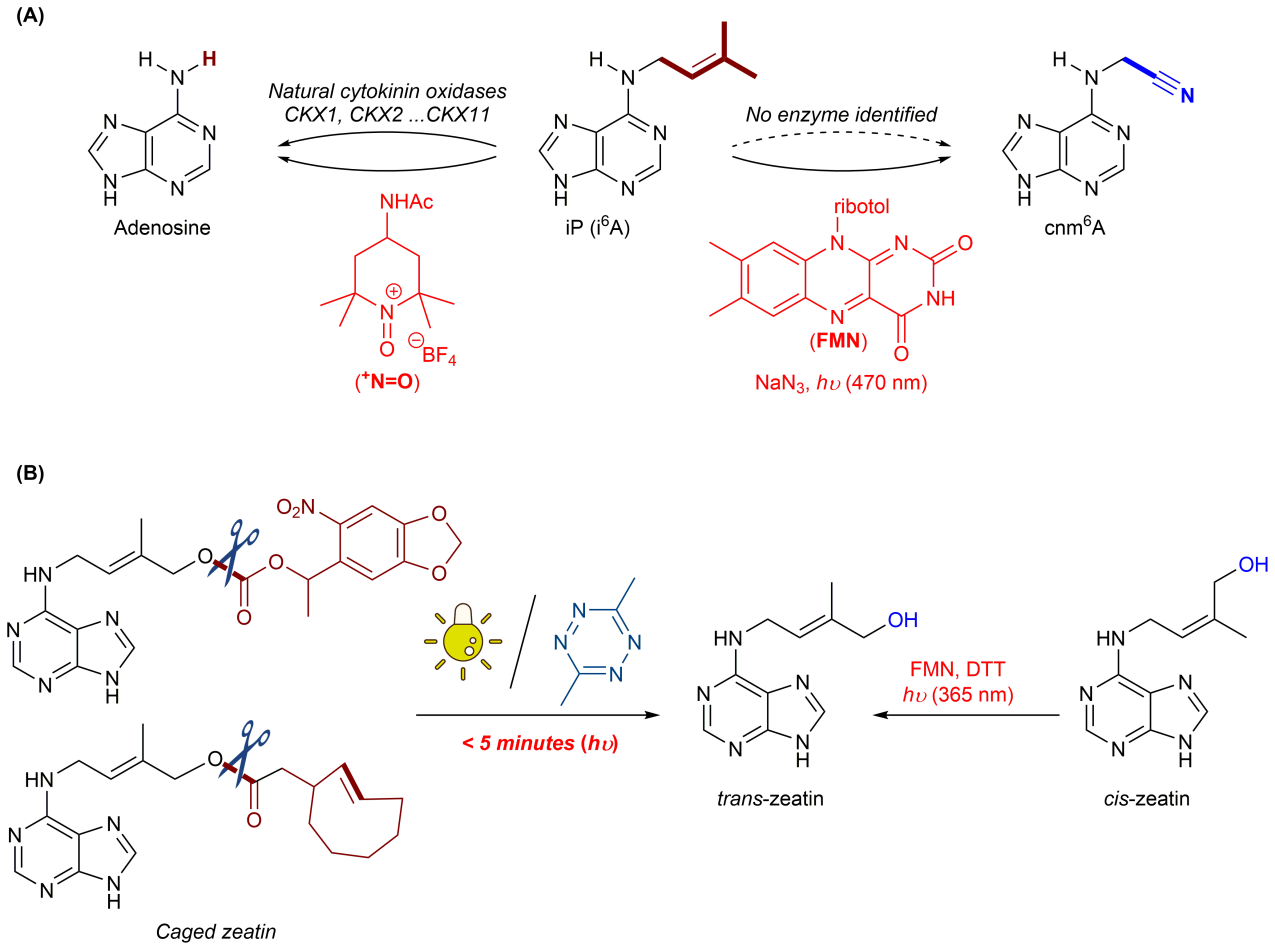
Chemical tools in cytokinins modulation. **(A)** Modulation of iP with *N*-oxoammonium salts (left) and FMN/NaN_3_/*hυ* (right). **(B)** Modulation of zeatin with photo-caged/small organic molecule-caged functionalities (left) and FMN/DTT/*hυ* (right).

Cytokinins (CKs) are adenine-derived compounds characterized by substitution at the *N* ^6^-position with either an isoprenoid or aromatic moiety. They play critical roles in plant growth and development, influencing processes such as seed germination, nutrient transport, flower and fruit development, and leaf senescence (Werner and Schmülling, 2009; Mok, 2019). Cytokinins exist primarily as active free bases (e.g., isopentenyladenine (iP), dihydrozeatin, *cis*-zeatin (cZ), and *trans*-zeatin (tZ)), which directly bind to cytokinin receptors, as well as in inactive conjugated forms (ribosides and nucleotides) (**Figure 1A**). Their concentration in plants is extremely low, typically in the pmol/g range of fresh weight. The occurrence, distribution, and variation of specific cytokinins depend on plant species, tissue type, and developmental stage. Interestingly, inactive cytokinin forms are often present in significantly higher concentrations compared to active free bases, suggesting tight regulation of active cytokinin levels to prevent uncontrolled signaling. This regulation is achieved through coordinated enzymatic processes involving cytokinin biosynthesis, modification, and degradation, largely governed by key enzymes such as isopentenyltransferase (IPT) for biosynthesis and cytokinin oxidase (CKX) for degradation, as well as by the expression of corresponding genes (IPT and CKX).

Given the crucial role of cytokinins in plant development, modifying their endogenous levels represents a promising approach for enhancing crop performance, especially under environmental stresses such as drought, heat, salinity, and heavy metal contamination. Genetic strategies, such as tissue-specific activation of isopentenyltransferase (IPT) genes, have successfully demonstrated spatial and temporal regulation of cytokinin synthesis, improving plant tolerance to abiotic stresses and delaying senescence, thereby enhancing crop yield potential (Qin et al., 2011; Nguyen et al., 2021). However, broad or constitutive expression of IPT often leads to undesired phenotypes, including impaired root growth and disrupted plant architecture, as reported early on by Smigocki et al. using constitutive promoters in maize and tobacco (Smigocki and Owens, 1988). Chemical modulation of cytokinin levels has emerged as a complementary alternative, providing improved specificity and temporal control without permanent genetic alterations. Several small-molecule regulators of cytokinin signaling and metabolism have been reported, including the cytokinin antagonist PI-55, which competitively inhibits cytokinin receptors, and INCYDE, an inhibitor of cytokinin oxidase/dehydrogenase (Spíchal et al., 2009; Nisler et al., 2010; Gemrotová et al., 2013; Prerostova et al., 2020). PI-55 and related antagonists like LGR-991 effectively decrease cytokinin perception, promoting root growth and stress resilience by mimicking conditions of low endogenous cytokinin status. In contrast, INCYDE application transiently elevates active cytokinin pools (e.g., *trans*-zeatin and *cis*-zeatin), enhancing heat tolerance and recovery when applied in combination with stress pre-acclimation treatments. Furthermore, caged cytokinins and photo-controlled chemical tools offer additional precision in cytokinin modulation, allowing rapid and reversible hormone activation in a highly localized manner. Nevertheless, chemical modulation also faces significant challenges. Achieving physiological concentrations of active cytokinins at precise temporal and spatial scales remains difficult.

Given these limitations, a pertinent question arises: *Could chemical methods be employed to regulate cytokinin levels to modulate plant growth and development effectively*? Chemical molecules offer distinct advantages, including precise temporal and spatial control. Additionally, selective manipulation of specific cytokinins can potentially be achieved through targeted chemical interventions. Moreover, given current limitations in gene-editing delivery systems, small molecule-based approaches present clear practical benefits. Nonetheless, the inherently low and dynamically fluctuating cytokinin concentrations pose significant challenges for direct chemical intervention at the whole-plant level (Jiang and Asami, 2018; Chen et al., 2025). To date, only a few successful examples have been reported, each with its own limitations (Cheng et al., 2020; Sun et al., 2023; Sun et al., 2024; Xie et al., 2024). Here, we will briefly introduce recent advancements over the past five years in chemical interventions targeting cytokinins and critically assess their strengths and weaknesses to guide future research efforts in this field.

## 2 The development of chemical tools and applications in cytokinins modulation

Cytokinin oxidase (CKX) specifically and irreversibly catalyzes the degradation of isopentenyl cytokinins, a process reliant upon its coenzyme, flavin adenine dinucleotide (FAD). Although the precise catalytic mechanism remains unclear, it is widely proposed that CKX interacts with cytokinins to form an imine intermediate, which is subsequently hydrolyzed to complete cytokinin degradation. Inspired by this mechanism, Cheng et al. recently developed a series of *N*-oxoammonium salts (^+^N=O) functioning as effective *artificial deprenylases* (Cheng et al., 2020). These chemical tools selectively remove the isopentenyl groups from cytokinins (e.g., iP and iPR) under mild conditions, displaying excellent biological compatibility without affecting other nucleosides, modifications, amino acids, or endogenous natural metabolites. Experiments indicated that *N*-oxoammonium salt possesses extremely low cytotoxicity, significantly reducing cellular iP and iPR levels across various cell lines. *Arabidopsis* seeds treated with salt exhibited accelerated germination, enhanced leaf growth, and increased root development, corresponding to substantially lowered iP and iPR concentrations *in vivo*. This study represents the first successful instance of chemically regulating plant growth by directly reducing cytokinin levels without disrupting their biosynthetic pathways, offering a powerful chemical biology tool for studying cytokinin function and holding considerable promise for crop improvement and breeding efficiency. On another front, despite significant advances over the past decade in identifying proteins associated with iP/iPR modification, artificial manipulation of these cytokinins into novel phytohormones has remained challenging, primarily due to the lack of appropriate bioorthogonal transformations targeting the prenyl group. To overcome this limitation, Xie et al. developed a visible-light-assisted bioorthogonal reaction employing flavin mononucleotide (FMN) and sodium azide under blue-light irradiation (Xie et al., 2024). This approach effectively mimicked enzymatic post-modification by selectively cleaving the prenyl double bond of iP/iPR, thereby generating an artificial *N* ^6^-cyanomethyl adenosine (cnm^6^A). Although significant incorporation of cnm^6^A was observed within tRNA, no free nucleoside form was detected in living cells. Notably, it exhibited reduced cytotoxicity compared to iPR, alleviating growth inhibition in poplar (*Populus trichocarpa*) suspension cells. This suggests that the hydrophilic cyanomethyl substituent introduced *via* chemical modification likely diminished binding affinity to hydrophobic receptor sites previously implicated in growth inhibition, potentially offering distinct and beneficial biological profiles.

Zeatin is a naturally occurring cytokinin in higher plants, known to regulate various physiological processes. Its biological activity is significantly influenced by structural modifications and reversible interconversion between its *cis*- and *trans*-isomers, with the *trans*-isomer exhibiting considerably stronger biological activity. Although the enzyme responsible for *cis*/*trans* isomerization of zeatin remains unidentified and the chemical basis of this conversion is still poorly understood, Sun et al. have recently advanced chemical strategies to modulate zeatin’s bioactivity by mimicking the plant’s natural regulation pathways (Sun et al., 2023) (**Figure 1B**). Utilizing photo-controlled bioorthogonal cleavage reactions and inverse electron-demand Diels-Alder reactions, they reversibly shielded active sites of zeatin derivatives, effectively blocking their metabolism by cytokinin oxidase (CKX) and glycosyltransferases (UGT). Upon exposure to specific stimuli, such as light or small molecules, these protecting groups were rapidly removed, restoring the physiological activity of zeatin in a highly controlled temporal and spatial manner. Additionally, Sun et al. introduced a UV-light-driven (365 nm) method utilizing FMN and dithiothreitol (DTT) to selectively control the *cis*/*trans* isomerization of zeatin’s prenyl side-chain under near-physiological aqueous conditions (Sun et al., 2024). Remarkably, this approach converted a substantial fraction of the less active *cis*-zeatin into its biologically potent *trans*-form. Moreover, the process was reversible, allowing temporary masking and restoration of cytokinin activity. Demonstrating practical applicability, this strategy successfully modulated seedling growth in rice by controlling *cis*/*trans* zeatin interconversion *in vivo*, providing valuable insights and powerful chemical tools for developing novel, intelligently responsive phytohormones aimed at enhancing agricultural productivity.

## 3 Discussion

Recent research has significantly advanced the field of cytokinin modulation using chemical tools. Notably, developments such as *N*-oxoammonium salts functioning as *artificial deprenylases* have enabled selective removal of isopentenyl groups from active cytokinins like iP and iPR, offering a means to reduce active cytokinin levels with high specificity. Additionally, photo-controlled bioorthogonal chemical reactions have emerged as powerful tools to temporally and spatially control zeatin activity by selectively interconverting its biologically active *trans*-isomer and inactive *cis*-isomer, allowing precise spatial and temporal control of cytokinin signaling. These innovations provide effective alternatives or complements to genetic approaches, enabling dynamic modulation of cytokinin activity without permanent alterations to endogenous biosynthetic pathways (**Table 1**).

**Table 1:** Summary of chemical tools for cytokinin modulation: mechanisms, advantages, limitations, and potential applications.

Chemical Tool	Mechanism of Action	Advantages	Limitations	Potential Applications
*N*-oxoammonium salts	Selective removal of isopentenyl groups from cytokinins (iP, iPR)	High specificity; low cytotoxicity; preserves other nucleotides	Limited field validation; challenges in plant uptake	Fine-tuned reduction of active cytokinin pools; control of germination and growth
FMN/azide visible-light bioorthogonal reaction	Cleavage of prenyl group double bond in i^6^A (iP/iPR); formation of cnm^6^A	Precise light control; highly selective chemical transformation	Efficiency and uptake in whole plants untested; limited to laboratory conditions	Development of synthetic cytokinin-like nucleosides; stress alleviation
Caged cytokinins	Inactivation of cytokinins *via* chemical caging; activated by light exposure	Temporal and spatial control; reversible activation	Requirement of external light source; possible incomplete activation	Studying localized cytokinin functions; targeted tissue activation
PI-55 (cytokinin receptor antagonist)	Competitive inhibition of cytokinin receptors (e.g., AHK4, AHK3)	Reduces cytokinin signaling; promotes root growth; stress tolerance	Potential off-target effects; partial receptor specificity	Regulation of root development; abiotic stress mitigation
INCYDE (cytokinin oxidase/dehydrogenase inhibitor)	Inhibition of CKX enzymes to elevate endogenous cytokinin levels	Enhances stress tolerance; boosts recovery after stress; easy application	Timing-sensitive effects; possible overstimulation of cytokinin responses	Improving heat and drought tolerance; enhancing plant recovery

However, despite these promising advancements, several crucial shortcomings persist that must be addressed before chemical cytokinin modulation can be effectively applied in real-world agricultural systems. Firstly, cytokinins naturally occur at very low concentrations (typically in the picomolar to nanomolar range) and exhibit rapid, dynamic changes depending on tissue type, developmental stage, and environmental conditions. This makes it inherently difficult to achieve consistent and physiologically relevant modulation of cytokinin levels using exogenous chemical agents (Cheng, 2025). Most studies to date have been conducted under controlled laboratory conditions, often using model organisms such as *Arabidopsis*. The translatability of these findings to crop species in complex, variable field environments remains largely untested. Second, and critically, one underrepresented aspect in current research is the challenge of plant uptake and in planta transport of bioactive small molecules. For any chemical modulator to function effectively in agricultural settings, it must not only be biologically active but also capable of being absorbed by plant tissues, translocated to the appropriate sites of action, and retained at effective concentrations. This becomes particularly crucial when the goal is spatiotemporal control of specific physiological responses. Without targeted delivery systems or tissue-specific transport strategies, even the most potent chemical modulators may have limited effectiveness or cause unintended systemic effects. To this end, future research should explore delivery technologies such as nanoparticle-based carriers, prodrug designs, or ligand-guided systems that could facilitate controlled release and targeted transport of synthetic cytokinins or their inhibitors to specific tissues or cell types. In addition, the long-term metabolic fate and environmental impact of chemically modified cytokinins remain poorly understood. Although existing tools generally exhibit low cytotoxicity and appear biologically compatible in controlled settings, their degradation pathways, persistence in soil or water systems, and potential to interfere with non-target organisms or signaling pathways have yet to be comprehensively studied. This presents a critical barrier to the safe and sustainable deployment of chemical cytokinin modulators in agriculture.

Looking ahead, several key directions should be prioritized. First, the development of more selective, sensitive, and stable molecules capable of modulating specific cytokinin types at physiologically relevant concentrations is essential. Responsive chemical systems – such as light-activated switches or environmentally triggered release mechanisms – could offer much-needed precision in field applications. Second, large-scale validation of these tools in economically important crops under real agricultural conditions is crucial to bridge the gap between lab and field. Third, integrated profiling techniques including metabolomics, transcriptomics, and high-resolution imaging can provide insights into the distribution, mode of action, and systemic impact of these chemical agents. Lastly, deeper mechanistic investigations into cytokinin receptor interactions and downstream signaling cascades at the molecular level will provide critical insights enabling rational chemical design and optimization, ultimately facilitating the development of practical, effective, and sustainable chemical strategies for agricultural improvement.
